# Short-Term Effects of Acupuncture on Open-Angle Glaucoma in Retrobulbar Circulation: Additional Therapy to Standard Medication

**DOI:** 10.1155/2011/157090

**Published:** 2011-03-07

**Authors:** Shin Takayama, Takashi Seki, Toru Nakazawa, Naoko Aizawa, Seri Takahashi, Masashi Watanabe, Masayuki Izumi, Soichiro Kaneko, Tetsuharu Kamiya, Ayane Matsuda, Akiko Kikuchi, Tomoyuki Yambe, Makoto Yoshizawa, Shin-ichi Nitta, Nobuo Yaegashi

**Affiliations:** ^1^Department of Traditional Asian Medicine, Graduate School of Medicine, Tohoku University, 1-1 Seiryo-machi, Aoba-ku, Sendai, Miyagi 980-8574, Japan; ^2^Department of Ophthalmology and Visual Science, Graduate School of Medicine, Tohoku University, Sendai 980-8574, Japan; ^3^Institute of Development, Aging and Cancer, Tohoku University, Sendai 980-8575, Japan; ^4^Research Division on Advanced Information Technology, Cyberscience Center, Tohoku University, Japan

## Abstract

*Background*. The relation between glaucoma and retrobulbar circulation in the prognosis has been indicated. *Purpose*. To investigate the effects of acupuncture on retrobulbar circulation in open-angle glaucoma (OAG) patients. *Methods*. Eleven OAG patients (20 eyes with OAG) who were treated by topical antiglaucoma medications for at least 3 months were enrolled. Acupuncture was performed once at acupoints BL2, M-HN9, ST2, ST36, SP6, KI3, LR3, GB20, BL18, and BL23 bilaterally. Retrobulbar circulation was measured with color Doppler imaging, and intraocular pressure (IOP) was also measured at rest and one hour after rest or before and after acupuncture. *Results*. The Δ value of the resistive index in the short posterior ciliary artery (*P* < .01) and the Δ value of IOP (*P* < .01) were decreased significantly by acupuncture compared with no acupuncture treatment. *Conclusions*. Acupuncture can improve the retrobulbar circulation and IOP, which may indicate the efficacy of acupuncture for OAG.

## 1. Introduction

Glaucoma is one of the causes of blindness [1] and the Tajimi Study showed that the prevalence of primary open-angle glaucoma (OAG) was 3.9% in Japan [2]. The main treatment strategy of glaucoma is to control the intraocular pressure (IOP) [3]. Although IOP reduction is currently the main target for the treatment of glaucoma, treatment modalities that enhance retrobulbar hemodynamics in addition to reducing IOP may have a beneficial effect on the glaucoma therapy. It has been reported that glaucoma is associated with reduction in the blood flow velocity and elevation of the resistive index (RI) in the retrobulbar vessels [4–7]. It has also been reported that patients with OAG have impaired hemodynamics in ophthalmic circulation [8–10]. The impaired ocular circulation contributes to the progression of glaucomatous damage [11–13]. Therefore, new drugs or interventions that improve ocular hemodynamics may be preferable.

Recently, acupuncture has been widely applied to treat several conditions such as neck pain, shoulder pain, lumbar pain, headache, and hypertension in Asian and Western countries, and it has also been found to be effective for many conditions in several randomized trials [14–20]. Acupuncture has also been used for the treatment of ocular diseases, including glaucoma, in traditional Chinese medicine [21]. We have shown that acupuncture therapy added to the standard medication could affect the IOP level in eyes with normal-tension glaucoma [22], and several other studies have demonstrated that acupuncture improves choroidal blood flow in the eye [23–25].

We have already reported that color Doppler imaging (CDI) by ultrasound is suitable for measuring the blood flow change in several organs during traditional Chinese medicine therapy [26–30]. The real-time and noninvasive hemodynamic measurement with CDI has been applied for measuring the retrobulbar vessel hemodynamics, and the reproducibility has already been shown [31]. In this study, we evaluate the hemodynamic changes in retrobulbar vessels by CDI to investigate the effect of acupuncture on OAG eyes.

## 2. Subjects

After the ethics committee approved the study, 11 patients diagnosed with OAG (20 eyes with OAG) were enrolled in this study. The patients received standard medical treatment for at least 3 months. The patients who had an experience of laser trabeculoplasty, any ocular surgery, or inflammation within the past year were excluded in the present study.

## 3. Methods

### 3.1. Acupuncture

On the trial days, the patients arrived under regular medications. They received acupuncture therapy as follows in the morning. The acupoints were selected on the basis of the principles of traditional Chinese medicine. Acupuncture was performed for 15 min using disposable stainless steel needles (0.16 mm or 0.20 mm × 40 mm; Seirin Co. Ltd., Shizuoka, Japan) at acupoints Cuanzhu (BL2), Taiyang (M-HN9), Sibai (ST2), Zusanli (ST36), Sanyinjiao (SP6), Taixi (KI3), and Taichong (LR3) bilaterally while the patient was in the supine position and at acupoints Fengchi (GB20), Ganshu (BL18), and Shenshu (BL23) bilaterally while the patient was in the prone position for 15 min. Each needle was simply inserted without any intention of eliciting specific responses (e.g., de-qi feelings) to a depth of approximately 20 mm at acupoints ST36, SP6, KI3, GB20, BL18, and BL23. For acupoints BL2, M-HN9, ST2, and LR3, the needles were inserted to a depth of approximately 3–10 mm. Neither needle manipulation techniques nor other auxiliary interventions were used. Five licensed acupuncturists and one physician-acupuncturist with over 5 years of acupuncture experience administered the acupuncture treatment.

### 3.2. Measurements

**Figure 1:**
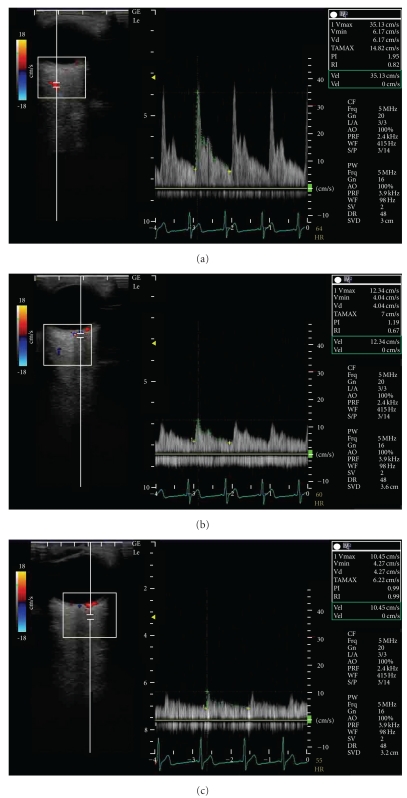
Horizontal scans by color Doppler imaging through the globe showing the (a) ophthalmic artery, (b) central retinal artery, and (c) short posterior ciliary artery.

 To minimize the effects of diurnal variation, all measurements were recorded at the same time of the day (between 10 AM and 11 AM) for each patient by the same examiner. As a control, the subjects received the measurements of the systemic hemodynamics, retrobulbar vessel hemodynamics, and IOP that were performed at rest and one hour after rest. One month later, they received the same measurements before and after acupuncture treatment. The systemic hemodynamics was measured by an oscillometer and the hemodynamics in retrobulbar vessels was measured by ultrasound (LOGIQ e, GE Healthcare, Tokyo, Japan). The ultrasound measurements were performed after 10-minute rest in an air-conditioned room, avoiding any pressure on the eye, with the patients in the supine position. CDI was performed with a 13 MHz linear transducer for retrobulbar vessels such as the ophthalmic artery (OA), central retinal artery (CRA), and short posterior ciliary artery (SPCA). The OA was examined approximately 20 mm behind the globe (Figure 1(a)), the CRA was examined within 5 mm of the retrolaminar portion of the optic nerve (Figure 1(b)), and the temporal SPCA was examined approximately 5–10 mm behind the globe (Figure 1(c)). All blood flow velocity waveforms were measured at the corrected Doppler angle. Resistive index (RI: (peak systolic velocity − end-diastolic velocity)/peak systolic velocity) was also measured in each retrobulbar vessel.

### 3.3. Statistical Analysis

Statistical analysis was performed with the SPSS software (version 16.0, SPSS Japan Inc., Tokyo, Japan). The parameters between before and after acupuncture or between control and acupuncture were compared by paired *t*-test.

## 4. Results

Table 1 shows the characteristics of the subjects. One male and ten female glaucoma patients with a mean age of 63 ± 11 years were observed. The systemic hemodynamic parameters including heart rate, blood pressure, and IOP are shown in Table 2. The blood pressure and heart rate did not change significantly by acupuncture.

The IOP level significantly decreased by acupuncture compared with before acupuncture (*P* < .05). The Δ value of IOP also significantly decreased by acupuncture compared with control (*P* < .01) (Table 2).

Retrobulbar vessel RI in the OA, CRA, and SPCA is shown in Table 3. The RI in the CRA and SPCA decreased significantly by acupuncture compared with before acupuncture (*P* < .05). The Δ value of RI in the SPCA also significantly decreased by acupuncture compared with control (*P* < .01) (Table 3).

## 5. Discussion

To our best knowledge, this is the first report on hemodynamic change in retrobulbar vessels related to acupuncture in OAG eyes. The present findings suggest that acupuncture can alter vessel resistance in the SPCA, even though the eyes are treated with standard medications.

The OA originates from the internal carotid artery. The CRA and SPCA are the ocular branches of the OA [32]. The CRA supplies blood to the retina and SPCA, to the choroid. CDI by ultrasound is useful for the measurement of the blood flow in various vessels in real time. Since it is impossible to determine the diameter of very small retrobulbar vessels, CDI cannot directly measure blood flow volume. However, the decrease of the distal vascular resistance in the SPCA indicates an increase of the blood flow in the choroid. We have already reported that acupuncture could increase the blood flow volume in the upper limb without an increase in the cardiac output, and the increased reaction in the blood flow was mediated by the decrease in the vascular resistance on the basis of the decreased vascular tone [30]. The mechanisms by which acupuncture can alter retrobulbar vessel circulation are still unclear. However, it has been reported that the blood flow in the eye is controlled by sympathetic and parasympathetic nerves, and it is related with the release of nitric oxide or calcitonin gene-related peptide [33, 34]; it has also been reported that the regulation of regional blood flow by somatic afferent stimulation is based on somatoautonomic reflex mechanisms in the choroidal blood flow of the eyeball [34]. The hemodynamic changes in the SPCA by acupuncture may be related with these mechanisms. Reduced blood flow velocities and increased vascular resistance in the retrobulbar arteries appear to be a risk factor for glaucoma progression [35–38]. Thus, acupuncture may be applied for additional therapy to treat OAG.

We should view these results cautiously because the present study was a case series study and intervention was provided only once. Longer observation of acupuncture therapy is needed to investigate the progression of glaucomatous damage associated with impaired ocular circulation.

## 6. Conclusions

The vessel resistance in the SPCA and the IOP level were decreased by acupuncture in OAG eyes. Acupuncture can affect the retrobulbar circulation and IOP despite the administration of standard medication. The present study implies the possibility that acupuncture is effective for OAG with standard medication. 

## Figures and Tables

**Table 1:** Characteristic data of the patients with open-angle glaucoma.

Variable	Value
Number of patients	11
Age (years)	63 ± 11
Sexuality (male, female)	(1, 10)
Number of eyes with glaucoma	20
Best corrected visual acuity	1.1 ± 0.3
Spherical equivalent (D)	−1.6 ± 3.2
Humphrey automated perimeter	
Mean deviation (dB)	−11.5 ± 7.8
Pattern standard deviation (dB)	10.2 ± 4.5
OCT RNFL thickness (*μ*m)	70.5 ± 21.8
The number of topical medications	
None	1
One kind	4
Two kinds	1
More than three kinds	5

**Table 2:** Blood pressure, heart rate, and intraocular pressure in control and acupuncture therapy. The values represent the mean and SD. *P < .05, **P < .01 versus rest or before acupuncture. ^†^ *P* < .05, ^††^ *P* < .01 versus control.

Parameter	Control			Acupuncture		
	Rest	After 1 hour	Δ value	Before	After	Δ value
Systole blood pressure (mm Hg)	116.4 ± 10.0	119.8 ± 7.6	3.4 ± 7.4	124.5 ± 12.9	122.6 ± 9.7	−1.1 ± 7.9
Diastolic blood pressure (mm Hg)	69.8 ± 6.5	68.6 ± 3.9	−1.0 ± 9.4	74.5 ± 5.4	72.0 ± 2.9	−3.0 ± 5.5
Heart rate (beats/min)	61.5 ± 7.3	60.1 ± 8.1	−2.5 ± 3.8	61.7 ± 8.5	60.3 ± 10.4	−2.4 ± 5.5
Intraocular pressure (mm Hg)	16.0 ± 4.1	17.1 ± 4.2**	1 ± 0.9	17.0 ± 5.0	16.0 ± 4.3*	−1 ± 1.9^††^

**Table 3:** Resistive index (RI) in the ophthalmic artery, central retinal artery, and short posterior ciliary artery. The values represent the mean and SD. *P < .05, **P < .01 versus before acupuncture. ^†^ *P* < .05, ^††^ *P* < .01 versus control.

Resistive index	Control			Acupuncture		
	Rest	After 1 hour	Δ value	Before	After	Δ value
Ophthalmic artery	0.74 ± 0.04	0.75 ± 0.05	0.006 ± 0.037	0.74 ± 0.04	0.74 ± 0.04	−0.006 ± 0.036
Central retinal artery	0.75 ± 0.09	0.72 ± 0.03	−0.027 ± 0.085	0.72 ± 0.05	0.68 ± 0.04*	−0.036 ± 0.059
Short posterior ciliary artery	0.68 ± 0.05	0.68 ± 0.04	0.004 ± 0.038	0.67 ± 0.04	0.64 ± 0.06*	−0.032 ± 0.054^††^
